# Comparative efficacies of the three echinocandins for *Candida auris* candidemia: real world evidence from a tertiary centre in India

**DOI:** 10.1093/mmy/myae065

**Published:** 2024-06-25

**Authors:** Parikshit S Prayag, Sampada A Patwardhan, Rasika S Joshi, Surabhi Dhupad, Tejashree Rane, Amrita P Prayag

**Affiliations:** Department of Infectious Diseases, Deenanath Mangeshkar Hospital, Pune, India; Department of Microbiology, Deenanath Mangeshkar Hospital, Pune, India; Department of Infectious Diseases, Deenanath Mangeshkar Hospital, Pune, India; Department of Clinical Pharmacology, Deenanath Mangeshkar Hospital, Pune, India; Department of Clinical Pharmacology, Deenanath Mangeshkar Hospital, Pune, India; Department of In house research, Deenanath Mangeshkar Hospital, Pune, India

**Keywords:** *Candida auris* candidemia, *Candida auris*, echinocandins

## Abstract

Though echinocandins are the first line of therapy for *C. auris* candidemia, there is little clinical data to guide the choice of therapy within this class. This was the first study to compare the three echinocandins in terms of efficacy and outcomes for *C. auris* candidemia. This was a retrospective analysis of 82 episodes of candidemia caused by *C. auris* comparing outcomes across the three echinocandins. Majority patients in our study were treated with micafungin. Susceptibility rates were the lowest for caspofungin (35.36% resistance), with no resistance reported for the other two echinocandins. When a susceptible echinocandin was chosen, caspofungin resistance was not a factor significantly associated with mortality. Also, when a susceptible echinocandin was used for therapy, the choice within the class did not affect clinical cure, microbiological cure, or mortality (*P* > 0.05 for all). Failure to achieve microbiological cure (*P* = 0.018) and receipt of immune-modulatory therapy (*P* = 0.01) were significantly associated with increased mortality. Significant cost variation was noted among the echinocandins. Considering the significant cost variation, comparable efficacies can be reassuring for the prescribing physician.

## Introduction

*Candida auris (C. auris)* is an important fungal pathogen causing nosocomial infections, especially in immunocompromised patients and in the intensive care unit (ICU) settings, and is associated with high mortality rates.^1^ The Centres for Disease Control and Prevention data showed that clinical cases of *C. auris* increased from 329 in 2018 to 1012 in 2021 in the United States.^2^ The burden of *C. auris* has been steadily increasing in India. A study of candidemia across multiple centres in India, published in 2015, showed that *C. tropicalis* was the predominant species.^3^ However, in this study, *C. auris* accounted for 5.3% of the total episodes of candidemia.^3^ In the same study, *C. auris* was found more in public hospitals than in private settings (8.2% vs. 3.9%).^3^ A study from western India published in 2022 showed that *C. auris* accounted for 43.03% of the 79 episodes of candidemia.^4^ This was the first study that showed *C. auris* as the predominant species causing candidemia.

Five different geographic clades have been described for *C. auris*.^5,6^ In a murine model of candidemia, the south Asian clade was associated with 80% mortality.^7^ Multidrug resistance is becoming an emerging problem in the management of infections caused by *C. auris*. Resistance rates are influenced by the type of clade and geographic locations. The south Asian clade, which is present in India, is associated with very high rates of resistance for fluconazole, as shown in the study published in western India.^4^ In the same study, amphotericin B resistance was 32.35%, and low rates of resistance were reported for the echinocandins.^4^

Based on in vitro data, echinocandins are considered to be the first line of therapy for candidemia caused by *C. auris*. However, there is very little data to guide the choice of therapy within the echinocandin class of drugs. A study published in 2020 found comparable efficacy between micafungin and anidulafungin for the treatment of candidemia; however, this study did not include patients in whom *C. auris* was the cause of candidemia.^8^ In the study published by Chakrabarti et al. in 2015, 7.7% of the isolates of *C. auris* were found to be resistant to caspofungin, while no resistance was found for micafungin or anidulafungin.^3^ The recommended method for antifungal susceptibility testing (AFST) is broth microdilution, which can be time-consuming and needs considerable technical expertise. This method is not uniformly available across the country, thus limiting the access of prescribing clinicians to accurate in vitro data. However, there are no comparative head-to-head studies for the three echinocandins, focusing solely on *C. auris*. There are differences in the safety profiles and costs of therapy of the three echinocandins, as well as the availability of these drugs across various parts of the country, and hence, it is important to have comparative data to empower physicians to choose the right echinocandin for *C. auris*. Our study aimed to analyse 82 episodes of candidemia caused by *C. auris*, comparing the three echinocandins, in terms of the in vitro efficacy and outcomes.

## Methods

### Patient selection

Adult patients with candidemia caused by *C. auris* (which was treated with echinocandin monotherapy) were selected for this retrospective analysis. Those patients who received an additional antifungal agent (in addition to the echinocandins), or those who received echinocandins for less than 72 hours were excluded from the final analysis. The institutional ethics committee granted us the permission to conduct this retrospective study. Only those patients in whom the drug being used was confirmed to be susceptible were included in the final analysis.

#### Blood cultures

Blood cultures were performed using the automated BD BACTEC Fx system (Becton Dickinson and Company, USA). Broth from positive blood culture vials was used to perform gram stain identification and was subcultured on Sabouraud’s Dextrose Agar (SDA) for further identification.

#### Species identification

Identification of *Candida* species was done by using the MALDI-TOF-MS (Matrix-Assisted Laser Desorption and Ionization-Time of Flight Mass Spectrometry) technology on the MALDI Biotyper Sirius (Bruker Daltonics Bremen, Germany).

#### AFST

Susceptibility testing was done using manual Broth Microdilution (BMD) method with Sensititre Y010 Panels (Thermo Fisher Scientific, USA) as per the manufacturer’s instructions.

#### Methods used for cost analysis and pharmacoeconomic analysis

The costs of echinocandins manufactured by different companies with the same formulations, strengths, and dosage forms were obtained from the pharmacy department of our hospital, and the data were analysed for cost variation. Cost range, cost ratio, and % cost variation were calculated per dose (per vial). The cost range was expressed as minimum cost per dose to maximum cost per dose. The cost ratio was calculated as the quotient of maximum cost over minimum cost. Percent cost variation per unit dosage for injectable formulations was calculated as follows: Cost variation (%) = [(Maximum cost − Minimum cost)/Minimum cost] ×100.

### Statistical analysis

The inter-group statistical comparison of distribution of categorical variables was done using χ^2^ test or Fisher’s exact probability, while the inter-group statistical comparison of distribution of means of continuous variables was done using analysis of variance

ANOVA with Post-Hoc Bonferroni’s test for multiple group comparisons. Multivariate logistic regression analysis with backward stepwise procedure was used to obtain the statistically significant and independent determinants of incidence of mortality. The underlying normality assumption was tested before subjecting the study variables to ANOVA. The entire data was statistically analysed using Statistical Package for Social Sciences (SPSS ver 24.0, IBM Corporation, USA) for MS Windows.

### Definitions used

#### Clinical cure

Clinical cure was defined as cessation of all antifungals and survival for at least 72 hours without the need to restart them.

#### Microbiological cure

Microbiological cure was defined as repeat blood cultures (sent after at least 3 days of appropriate antifungal therapy) showing no growth of *C. auris*.

## Results

A total of 82 episodes of *C. auris* candidemia were included in the final analysis. In our study, micafungin was the commonest echinocandin (*n* = 55, 67.1%) used for treating episodes of candidemia caused by *C. auris*. This was followed by caspofungin (*n* = 15, 18.3%) and then by anidulafungin (*n* = 12, 14.6%).

Table 1 shows the clinical and demographic characteristics of the study population. Distribution of demographic characteristics such as mean age did not differ significantly across three antifungal therapy groups (*P*-value > 0.05 for all). Significantly higher proportion of cases in the micafungin group were males, compared to the anidulafungin group (*P*-value < 0.05). Distribution of co-morbidities and risk factors did not differ significantly across three antifungal therapy groups, except a significantly higher proportion of cases in the anidulafungin group were on total parenteral nutrition, compared to the caspofungin group (*P*-value < 0.05). A significantly higher proportion of cases in Group C [Anidulafungin] had a higher prevalence of chronic liver disease (CLD) compared to the cases in Group A [Micafungin] and Group B [Caspofungin] (*P*-value < 0.05 for both).

**Table 1.** Inter-antifungal therapy group comparisons of demographic and other clinical characteristics, including treatment history among the cases studied.

		Group A [Micafungin]	Group B [Caspofungin]	Group C [Anidulafungin]	*P*-value (Inter-group)		
Variables		(*n* = 55)	(*n* = 15)	(*n* = 12)	A vs. B	A vs. C	B vs. C
Age (years)	Mean ± SD	53.55 ± 15.45	46.33 ± 16.76	55.64 ± 17.76	0.378^NS^	0.999^NS^	0.441^NS^
Sex, *n* (%)	Male	44 (80.0%)	12 (80.0%)	5 (41.7%)	0.999^NS^	0.017*	0.065^NS^
	Female	11 (20.0%)	3 (20.0%)	7 (58.3%)			
DM, *n* (%)	Yes	24 (43.6%)	5 (33.3%)	4 (33.3%)	0.999^NS^	0.999^NS^	0.999^NS^
	No	31 (56.4%)	10 (66.7%)	8 (66.7%)			
CKD, *n* (%)	Yes	6 (10.9%)	2 (13.3%)	3 (25.0%)	0.999^NS^	0.606^NS^	0.999^NS^
	No	49 (89.1%)	13 (86.7%)	9 (75.0%)			
CLD, *n* (%)	Yes	1 (1.8%)	0	3 (25.0%)	0.999^NS^	0.002**	0.006**
	No	54 (98.2%)	15 (100.0%)	9 (75.0%)			
Malignancy, *n* (%)	Yes	6 (10.9%)	2 (13.3%)	1 (8.3%)	0.999^NS^	0.999^NS^	0.999^NS^
	No	49 (89.1%)	13 (86.7%)	11 (91.7%)			
Neutropenia, *n* (%)	Yes	2 (3.6%)	1 (6.7%)	0	0.999^NS^	0.999^NS^	0.999^NS^
	No	53 (96.4%)	14 (93.3%)	12 (100.0%)			
Pyelonephritis, *n* (%)	Yes	2 (3.6%)	0	0	0.999^NS^	0.999^NS^	0.999^NS^
	No	53 (96.4%)	15 (100.0%)	12 (100.0%)			
Abdominal surgery, *n* (%)	Yes	8 (14.5%)	1 (6.7%)	2 (16.7%)	0.999^NS^	0.999^NS^	0.999^NS^
	No	47 (85.5%)	14 (93.3%)	10 (83.3%)			
Colonization with candida, *n* (%)	Yes	8 (14.5%)	1 (6.7%)	1 (8.3%)	0.999^NS^	0.999^NS^	0.999^NS^
	No	47 (85.5%)	14 (93.3%)	11 (91.7%)			
TPN, *n* (%)	Yes	7 (12.7%)	0	4 (33.3%)	0.581^NS^	0.168^NS^	0.035*
	No	48 (87.3%)	15 (100.0%)	8 (66.7%)			
Other immunomodulatory therapy, *n* (%)	Yes	8 (14.5%)	5 (33.3%)	4 (33.3%)	0.341^NS^	0.442^NS^	0.999^NS^
	No	47 (85.5%)	10 (66.7%)	8 (66.7%)			
Steroids, *n* (%)	Yes	20 (36.4%)	4 (26.7%)	3 (25.0%)	0.999^NS^	0.999^NS^	0.999^NS^
	No	35 (63.6%)	11 (73.3%)	9 (75.0%)			
Central line, *n* (%)	Yes	33 (60.0%)	8 (53.3%)	9 (75.0%)	0.999^NS^	0.999^NS^	0.779^NS^
	No	22 (40.0%)	7 (46.7%)	3 (25.0%)			
DTP	Yes	3 (42.9%)	2 (100.0%)	2 (50.0%)	0.604^NS^	0.999^NS^	0.881^NS^
	No	4 (57.1%)	0	2 (50.0%)			
Prior Azole therapy	Yes	22 (40.0%)	4 (26.7%)	5 (41.7%)	0.885	0.999^NS^	0.999^NS^
	No	33 (60.0%)	11 (73.3%)	7 (58.3%)			
Duration of antifungal therapy (days)	Mean ± SD	15.00 ± 7.06	16.00 ± 9.36	11.58 ± 6.57	0.999^NS^	0.999^NS^	0.999^NS^
Echocardiography done	Yes	42 (76.4%)	10 (66.7%)	8 (66.7%)	0.446 ^NS^	0.484 ^NS^	1.000 ^NS^
	No	13 (23.6%)	5 (33.3%)	4 (33.3%)			
Fundoscopy done	Yes	35 (63.6%)	10 (66.7%)	6 (50.0%)	0.828 ^NS^	0.379 ^NS^	0.381 ^NS^
	No	20 (36.4%)	5 (33.3%)	6 (50.0%)			

*P*-value for age and duration of antifungal by ANOVA with Bonferroni’s Post-Hoc test for multiple group comparisons, the rest of the *P*-values by χ^2^ test. *P*-value < 0.05 is considered to be statistically significant. *P-value < 0.05, **P-value < 0.01, NS—Statistically non-significant.

DM: Diabetes Mellitus, CKD: Chronic Kidney Disease, CLD: Chronic Liver Disease, TPN: Total Parental Nutrition, DTP: differential time to positivity

Minimal inhibitory concentration (MIC) 50 (concentration of the echinocandin that would inhibit 50% of the fungal isolates) and MIC 90 (concentration of the echinocandin that would inhibit 90% of the fungal isolates), as well as the percentage resistance for each of the echinocandins, against the 82 isolates of *C. auris* tested by using the manual broth microdilution method were recorded. The MIC90 values were found to be higher for caspofungin (MIC90 = 8.0 μg/ml, % resistance = 35.36) as compared to the other two echinocandins (MIC90 = 0.5 μg/ml, % resistance = 0 for both). The MIC50 values did not differ drastically between the three echinocandins (caspofungin and anidulafungin MIC50 = 0.25 μg/ml, micafungin MIC50 = 0.12 μg/ml).

Table 2  shows that the distribution of outcome variables such as incidence of clinical and microbiological cure, incidence of mortality, and level of SOFA score did not differ significantly across three antifungal therapy groups (*P*-value > 0.05 for all).

**Table 2.** Inter-antifungal therapy group comparisons of various outcome variables.

		Group A [Micafungin]	Group B [Caspofungin]	Group C [Anidulafungin]	*P*-value		
Outcome variables		(*n* = 55)	(*n* = 15)	(*n* = 12)	A vs. B	A vs. C	B vs. C
Clinical cure, *n* (%)	Yes	39 (70.9%)	11 (73.3%)	8 (66.7%)	0.853^NS^	0.771^NS^	0.706^NS^
	No	16 (29.1%)	4 (26.7%)	4 (33.3%)			
Microbiological cure, *n* (%)	Yes	36 (83.7%)	7 (87.5%)	4 (80.0%)	0.999^NS^	0.999^NS^	0.999^NS^
	No	7 (16.3%)	1 (12.5%)	1 (20.0%)			
Day 28 mortality, *n* (%)	Death	16 (29.1%)	4 (26.7%)	7 (58.3%)	0.999^NS^	0.158^NS^	0.251^NS^
	Survived	39 (70.9%)	11 (73.3%)	5 (41.7%)			
SOFA score, *n* (%)	0–6	35 (63.6%)	11 (73.3%)	8 (66.7%)	0.999^NS^	0.999^NS^	0.999^NS^
	7–9	10 (18.2%)	3 (20.0%)	3 (25.0%)			
	10–12	9 (16.4%)	0	0			
	>12	1 (1.8%)	1 (6.7%)	1 (8.3%)			

P-value by χ^2^ test. *P*-value < 0.05 is considered to be statistically significant. NS—Statistically non-significant.

As shown in Table 3, the distribution of incidence of mortality did not differ significantly between groups of cases with different age groups and groups of male and female cases studied (*P*-value > 0.05 for all). Distribution of incidence of mortality was significantly higher in the diabetes mellitus group, the group of cases requiring other immune-modulatory therapy (apart from steroids), and the group of patients who did not achieve microbiological cure (*P*-value < 0.05 for all). The incidence of mortality was significantly higher in the group of cases with no abdominal surgery compared to the group of cases with the abdominal surgery (*P*-value < 0.05). The incidence of mortality did not differ significantly across various clinical characteristics such as presence of chronic kidney disease (CKD), malignancy, neutropenia, pyelonephritis, prior colonization with candida, requirement of total parental nutrition (TPN), receipt of steroids, presence of central line, patients with catheter-related blood stream infection (CRBSI), prior azole therapy, the choice of echinocandin, and caspofungin resistance (as long as a susceptible echinocandin was chosen) (*P*-value > 0.05 for all).

**Table 3.** Univariate statistical analysis showing the determinants of incidence of mortality.

		Mortality						
		Death (*n* = 27)		Survived (*n* = 55)		Total (*n* = 82)		
Variables		*n*	%	*n*	%	*n*	%	*P*-value
Age group (years)	<50	8	25.0	24	75.0	32	100.0	0.269^NS^
	≥50	19	38.0	31	62.0	50	100.0	
Sex	Male	18	29.5	43	70.5	61	100.0	0.262^NS^
	Female	9	42.9	12	57.1	21	100.0	
DM	Yes	15	45.5	18	54.4	33	100.0	0.048*
	No	12	24.5	37	75.5	49	100.0	
CKD	Yes	6	54.5	5	45.5	11	100.0	0.101^NS^
	No	21	29.6	50	70.4	71	100.0	
CLD	Yes	1	25.0	3	75.0	4	100.0	0.999^NS^
	No	26	33.3	52	66.7	78	100.0	
Malignancy	Yes	2	22.2	7	77.8	9	100.0	0.711^NS^
	No	25	34.2	48	65.8	73	100.0	
Neutropenia	Yes	2	66.7	1	33.3	3	100.0	0.251^NS^
	No	25	31.6	54	68.4	79	100.0	
Pyelonephritis	Yes	1	50.0	1	50.0	2	100.0	0.999^NS^
	No	26	32.5	54	67.5	80	100.0	
Abdominal surgery	Yes	0	0.0	11	100.0	11	100.0	0.013*
	No	27	38.0	44	62.0	71	100.0	
Colonization with candida	Yes	4	40.0	6	60.0	10	100.0	0.723^NS^
	No	23	31.9	49	68.1	72	100.0	
TPN	Yes	4	36.4	7	63.6	11	100.0	0.999^NS^
	No	23	32.4	48	67.6	71	100.0	
Other immunumodulatory therapy	Yes	10	58.8	7	41.2	17	100.0	0.011*
	No	17	26.2	48	73.8	65	100.0	
Steroids	Yes	12	44.4	15	55.6	27	100.0	0.120^NS^
	No	15	27.3	40	72.7	55	100.0	
Central line	Yes	18	36.0	32	64.0	50	100.0	0.459^NS^
	No	9	28.1	23	71.9	32	100.0	
DTP	Yes	3	42.9	4	57.1	7	100.0	0.999^NS^
	No	3	50.0	3	50.0	6	100.0	
Prior Azole therapy	Yes	10	32.3	21	67.7	31	100.0	0.920^NS^
	No	17	33.3	34	66.7	51	100.0	
Directed therapy	Micafungin	16	29.1	39	70.9	55	100.0	0.126^NS^
	Caspofungin	4	26.7	11	73.3	15	100.0	
	Anidulafungin	7	58.3	5	41.7	12	100.0	
Microbiological cure	Yes	11	23.4	36	76.6	47	100.0	0.017*
	No	6	66.7	3	33.3	9	100.0	
Caspofungin resistance	Yes	8	27.6	21	72.4	29	100.0	0.440^NS^
	No	19	35.9	34	64.1	53	100.0	

*P*-value by χ^2^ test. *P*-value < 0.05 is considered to be statistically significant. *P-value < 0.05, NS—Statistically non-significant.

The incidence of mortality was independently and significantly associated with the probable determinants such as requirement of other immunomodulatory therapy and failure to achieve microbiological cure after adjusting for various clinical characteristics such as age, sex, other comorbidities, requirement of antifungal therapy etc. (*P*-value < 0.05 for all) (Table 4).

**Table 4.** Multivariate logistic regression analysis to find out the independent determinants of incidence of mortality [backward stepwise procedure].

Risk factors (variables in the model)		Odds ratio (OR)	95% CI for odds ratio	*P*-value
Other immunomodulatory therapy	Absent	1.00	–	–
	Present	10.129	1.379–74.423	0.023*
Microbiological cure	Present	1.00	–	–
	Absent	14.386	2.007–103.091	0.008**

[Odds Ratio = 1: Reference Category]. Dependent variable: Mortality. **P-value < 0.01, NS—Statistically non-significant.

Other variables in the model: Age, sex, CKD, CLD, Malignancy, Neutropenia, Pyelonephritis, Abdominal surgery, Colonization with candida, TPN, Steroids, Central line, DTP, Prior Azole therapy, and directed therapy.

Table 5 shows a comparative cost analysis of the echinocandins and the relevant pharmacoeconomic data.

**Table 5.** Cost analysis with respect to the echinocandins.

Drug	Formulations	Dose (mg)	Manufacturing companies	Cost range (INR)	Cost ratio	Cost variation (%)
Micafungin	1	100	11	2493–8999	3.6	260.97
Anidulafungin	1	100	11	5950–12 700	2.13	113.4
Caspofungin	1	70	10	4200–16 000	3.8	280.95
		50	10	3999–16 240	4.06	306.1

## Discussion

In recent years, *C. auris* has been reported to be a major cause of invasive fungal infections worldwide. Variable antifungal susceptibility profiles and the development of resistance to different classes of antifungal drugs pose challenges in their treatment. Based on in vitro data, the echinocandins have emerged as the drugs of choice for treating candidemia caused by *C. auris*. There are many common features of the three echinocandins. They are available exclusively as intravenous formulations. These are drugs that are non-dialyzable and do not need dose modification in patients with renal impairment. They do not effectively penetrate into certain body sites (such as the lower genitourinary tract, central nervous system). These are drugs with high molecular weights and considerable protein binding. However, the three echinocandins available (micafungin, anidulafungin, and caspofungin) also exhibit some variations. The metabolism of each of these drugs in the body is different. Caspofungin is metabolized in the liver by hydrolysis and N-acylation,^9^ micafungin is metabolized by arylsulfatase, catechol-O-methyltransferase, and several cytochrome P450 (CYP) isoenzymes,^10^ while anidulafungin is non-enzymatically metabolized.^11^ The concerns for interactions are higher with caspofungin.

There are very few studies that compare the efficacy of the echinocandins for candidemia. A randomized, double-blinded trial comparing micafungin and caspofungin for candidemia published in 2007 showed no statistically significant difference between the therapies as far as treatment success was concerned.^12^ This study, however, did not include any patients with *C. auris* candidemia. A retrospective cohort study published in 2020 found similar clinical efficacy as well as safety for micafungin as well as anidulafungin.^8^ Again, this study did not have patients with *C. auris* candidemia. There are no well-designed studies comparing the efficacy of the three echinocandins for *C. auris* candidemia, a problem of increasing magnitude in our settings.

In our study, we studied 82 patients with candidemia caused by *C. auris*. As shown, the baseline characteristics were comparable. The anidulafungin group contained more patients with CLD than the other groups. Though studies have demonstrated no significant difference in the hepatotoxicity associated with the different echinocandins,^13^ anidulafungin has been traditionally thought of as a safer drug from a hepatic standpoint, due in part to the fact that it is not metabolized in the liver. Also, a study published in 2015 suggested that switching from caspofungin to anidulafungin in cancer patients with deranged hepatic function led to an improvement in the liver function tests (LFTs).^14^ This has led to a ‘channelling bias’ where the patients with deranged LFTs tend to be treated more frequently with anidulafungin. This was the same pattern observed in our analysis, with caspofungin not being chosen for any of the patients with deranged LFTs.

In this study, the MIC90 values were found to be higher for caspofungin. Also, the susceptibility rates were the lowest for caspofungin with no resistance reported for the other two echinocandins. In the study published by Chakrabarti et al. in 2015, 7.7% of the *C. auris* isolates collected from various hospitals across India, were found to be resistant to caspofungin, with no resistance reported for anidulafungin or micafungin [3]. A large multicenter study from India that analysed the antifungal susceptibility rates of 350 isolates of *C. auris* found the MIC90 values (mg/l) for caspofungin, anidulafungin, and micafungin to be 2.0, 1.0, and 0.25, respectively.

In our study, a higher incidence of caspofungin resistance was found, with a wide gap between the MIC50 and MIC90 values. Caspofungin is known to be associated with the Eagle effect (paradoxical growth of the wild-type strain at higher concentrations of the drug). This, however, may not be reflected as in vivo resistance.^15^ Eagle effect is not a result of genetic mutations and is associated with adaptive changes of the organism with cell wall modifications in response to environmental stress. Caspofungin is known to be associated with a high degree of interlaboratory variation, owing to which EUCAST does not recommend in vitro susceptibility testing for caspofungin. However, we selected only those patients who were treated with an echinocandin confirmed to be susceptible by AFST. Also, we did not find any difference in the MIC90 values for anidulafungin and micafungin. Furthermore, as long as a susceptible echinocandin was chosen, caspofungin resistance was not associated with increased mortality in our study. Further, larger studies are needed to study whether higher MICs translate into worse outcomes as far as caspofungin is concerned.

As shown in our study, 27 of the 82 patients did not survive at day 28, and this shows the high mortality in patients with *C. auris* candidemia. The overall clinical and microbiological cure was similar for patients treated with either of the three echinocandins. A higher proportion of the patients treated anidulafungin and micafungin had Sequential Organ Failure Assessment (SOFA) scores > 6. This shows that the treating physicians preferred these drugs in patients who were more critically ill. The choice of echinocandin used for directed therapy was not associated with mortality on the univariate or multivariate analyses. Instead, on multivariate analysis, receipt of immunomodulatory therapy and failure to achieve microbiological cure were associated with higher mortality. Thus, in this study, the underlying patient profile and microbiological cure influenced the mortality rates rather than the choice of echinocandin. This is a significant finding and can be used to determine the choice of echinocandin within our settings. It also reinstates the importance of sending repeat blood cultures and ensuring microbiological clearance in these patients.

All echinocandins are given exclusively by the intravenous route. In general, echinocandins are expensive drugs. A study from Australia comparing the economics of micafungin and caspofungin in the treatment of candidemia found that Micafungin was cost-equivalent to caspofungin in treating candidemia.^16^ In the Indian settings, cost is often an important factor when it comes to choosing within the echinocandin class. As shown in Table 5, there is significant variation amongst the echinocandins in India. It is often difficult to determine the most expensive among the three; this is subject to the variations in brands available in a particular center. Given the high prices and significant cost variation, comparable efficacies can be reassuring for the prescribing physician.

Overall, our study uniquely demonstrates that the outcomes in the different groups of patients with candidemia caused by *C. auris* receiving the three echinocandins may be comparable, when a susceptible echinocandin is used for therapy. Larger studies and randomized control trials are needed to compare the efficacy of the available echinocandins, so that physicians can choose the drug freely based on availability and economic constraints, especially in resource-limited settings. Also, there are newer therapeutic options on the horizon, and hence, larger comparative studies are needed.^17^

### Limitations

Our study has certain limitations. The number of episodes studied was relatively small, though this is the only study comparing the three echinocandins in the setting of candidemia caused by *C. auris*. Also, we used the commercial panels for performing broth microdilution testing. Though the accuracy of this test has been determined to be comparable to manual broth microdilution, the latter still remains the reference method. Also, sicker patients in our study were more likely to be treated with micafungin or anidulafungin, as against caspofungin. However, the differences were not statistically significant. Another important consideration is the fact that caspofungin has been found to be inactive against *C. auris* biofilms, and hence, further larger studies should explore the comparative efficacy of the echinocandins in such settings, as these patients were relatively underrepresented in our study.^18^

## Conclusions

To the best of our knowledge, this is the first study that compares the efficacy of the three echinocandins in the setting of *C. auris* candidemia. In our study, though micafungin and anidulafungin had better in vitro efficacy, the clinical efficacy of the three echinocandins was found to be comparable for treating patients with *C. auris* candidemia, when a susceptible echinocandin was chosen. This is an important finding given the wide variations in the cost and availability of echinocandins. Larger studies are needed to study the comparative efficacy of the three echinocandins for helping the clinicians better manage this increasingly complex fungal infection.

## Supplementary Material

myae065_Supplemental_Files
