# Supplementary material for: Comparative efficacies of the three echinocandins for Candida auris candidemia: real world evidence from a tertiary centre in India
Source: Med Mycol. 2024 Jun 25;62(7):myae065. doi: 10.1093/mmy/myae065 (PMC11250272; doi:10.1093/mmy/myae065)
Supplement: myae065_Supplemental_Files [file myae065_supplemental_files.zip › mm-2024-0087-File007.docx]

| **Directed therapy** | **No. of cases** | **% of cases** |
| --- | --- | --- |
| Micafungin | 55 | 67.1 |
| Caspofungin | 15 | 18.3 |
| Anidulafungin | 12 | 14.6 |
| Total | 82 | 100.0 |

**Supplement Table a) Distribution of candidemia cases treated with different antifungal therapy.**
