# Supplementary material for: Comparative efficacies of the three echinocandins for Candida auris candidemia: real world evidence from a tertiary centre in India
Source: Med Mycol. 2024 Jun 25;62(7):myae065. doi: 10.1093/mmy/myae065 (PMC11250272; doi:10.1093/mmy/myae065)
Supplement: myae065_Supplemental_Files [file myae065_supplemental_files.zip › mm-2024-0087-File008.docx]

| **Echinocandin** | **MIC50 (μg/ml)** | **MIC90 (μg/ml)** | **% Resistance** |
| --- | --- | --- | --- |
| Micafungin | 0.12 | 0.5 | 0 |
| Anidulafungin | 0.25 | 0.5 | 0 |
| Caspofungin | 0.25 | 8.0 | 35.36 |

**Supplement Table b) MIC50, MIC90 and percentage resistance for the three echinocandins**

***MIC: Minimal inhibitory concentration**
